# Characterisation of the expression and function of the GM-CSF receptor α-chain in mice

**DOI:** 10.1002/eji.200636892

**Published:** 2007-09

**Authors:** Marcela Rosas, Siamon Gordon, Philip R Taylor

**Affiliations:** 1 Medical Biochemistry and Immunology, Cardiff University School of MedicineCardiff, UK; 2 Sir William Dunn School of Pathology, University of OxfordOxford, UK

**Keywords:** Cell proliferation, Cytokines, Inflammation, Macrophages, Neutrophils

## Abstract

The granulocyte-macrophage colony-stimulating factor (GM-CSF) is a hematopoietic cytokine able to regulate a variety of cell functions including differentiation of macrophages and granulocytes, dendritic cell development and the maintenance of homeostasis. It binds specifically to its receptor, which is composed of a cytokine-specific α-chain (GM-CSF receptor α-chain, GMRα) and a β-chain shared with the receptors for interleukin-3 and interleukin-5. In this report, we present a comprehensive study of GMRα in the mouse. We have found that the mouse GMRα is polymorphic and alternatively spliced. In the absence of specific antibodies, we generated a novel chimeric protein containing the Fc fragment of human IgG1 coupled to mouse GM-CSF, which was able to specifically bind to GMRα and induce proliferation of GMRα-transduced Ba/F3 cells. We used this reagent to perform the first detailed FACS study of the surface expression of mouse GMRα by leucocytes. Highest expression was found on monocytes and granulocytes, and variable expression on tissue macrophages. The GM-CSF receptor in mice is specifically expressed by myeloid cells and is useful for the detection of novel uncharacterised myeloid populations. The ability to detect GM-CSF receptor expression in experimental studies should greatly facilitate the analysis of its role in immune pathologies.

## Introduction

The granulocyte-macrophage colony-stimulating factor (GM-CSF) was first isolated from mouse lung cell-conditioned media as a small glycoprotein (24–33 kDa) able to generate both macrophage and granulocyte colonies from mouse bone marrow precursor cells [1]. However, a variety of studies have shown other relevant functions including its capacity to enhance host defense mechanisms against bacterial and fungal infection [1], its contribution to dendritic cell development [2], and its involvement in the catabolism of pulmonary surfactant to maintain homeostasis in the alveoli of the lung [3]. GM-CSF can modulate effector functions not only on myeloid cells but also on other cell types expressing the GM-CSF receptor including type II alveolar epithelial cells [4], CD34^+^ progenitor cells [5], uterine cells [6], vascular endothelial cells [7], [8] and fibroblasts [9].

GM-CSF binds specifically to its receptor, which is composed of a cytokine-specific α-chain (GM-CSF receptor α chain, GMRα) and β-chain (βc), which is shared with the receptors for interleukin (IL)-3 and IL-5 [10]. It associates with GMRα with low affinity and rapid dissociation kinetics, but the formation of the αβ heterodimeric complex mediates a stable interaction with high affinity and slow dissociation kinetics. Besides enhancing cytokine binding, the βc plays a key role in signal transduction, which can also be modulated by IL-3 and IL-5 [11], [12] In the mouse, there are two homologous βc, AIC2A and AIC2B. IL-3 can induce the formation of the αβ complex using either AIC2A or AIC2B, but only AIC2B is able to create the αβ complex for GM-CSF and IL-5 [13], [14].

Molecular cloning of the mouse GMRα cDNA revealed a single transmembrane polypeptide of 387 amino acids with relatively low homology to its human counterpart (less than 35%). It is a type I membrane protein subdivided into three regions: an extracellular domain, a transmembrane region, and a cytoplasmic domain. The extracellular domain shares conserved structural features of the cytokine receptor superfamily containing three fibronectin type III (FN-III)-like domains, four cysteine residues involved in heterodimerization of the receptor, and an imperfect WSXWS box (WGEWS), which is required for protein folding and cell surface receptor binding [11], [15]. The cytoplasmic domain is short and characterised by the presence of a highly conserved proline-rich domain (PPXP), which is adjacent to the plasma membrane and involved in signal transduction (reviewed in [10]).

Several studies on the human GMRα have demonstrated that as consequence of alternative splicing or metalloproteinase activity, GMRα loses its tramsmembrane domain resulting in a soluble isoform, which is able to antagonise GM-CSF binding and function [16–19]. However, alternatively spliced isoforms of the murine receptor have not been reported [15].

In spite of the obvious importance of GM-CSF and the wide use of mice in immunological studies there are no useful reagents for the detection of the mouse GMRα or extensive studies of its expression and function. In this report, we present a comprehensive study of GMRα in the mouse. We have found that the mouse GMRα is polymorphic and alternatively spliced. Due to the restricted availability of specific antibodies against the extracellular domain of GMRα, we coupled the mouse GM-CSF to the Fc fragment derived from human IgG (GM-Fc) to characterise surface expression of the mouse GMRα on distinct leucocyte populations. GM-Fc is able to bind and activate the receptor, and thereby, provides a novel way to examine the regulation and expression of GMRα during steady state and immunological challenge.

## Results

### The mouse GM-CSF receptor is alternatively spliced and polymorphic

**Figure 1:**
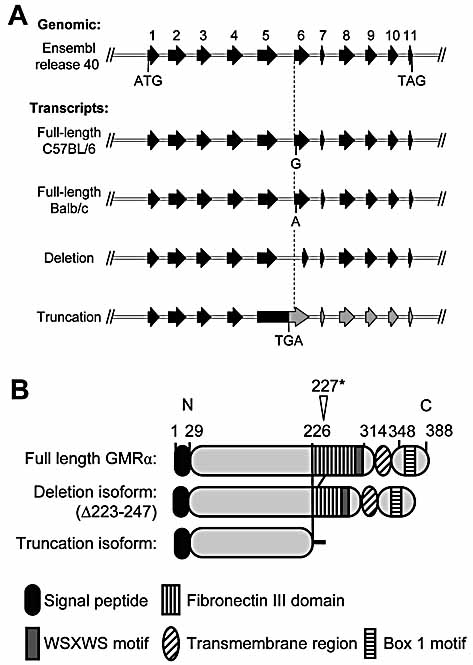
Characterisation of GMRα in the mouse model. (A) Schematic representation of the 11 exons coding for GMRα. The recently published sequence (Ensembl release 40) corresponds to the full-length sequence of C57BL/6 mice whereas Balb/c mice display a polymorphic change in exon 6. We observed two additional splice forms, one with a small deletion and a truncated form, the changes in which are shown schematically. Black arrows indicate coding exons while grey arrows indicate non-coding sequence. (B) Schematic representation of the three predicted isoforms of the mouse GMRα. The full-length isoform is composed of a short cytoplasmic tail, a transmembrane region and an extracellular region, which contains an FN-III domain where there is a polymorphic change at residue 227* (Asp for Balb/c and Gly for C57BL/6). The deletion transcript results in an in-frame deletion in the FN-III domain, but retains a transmembrane domain. The truncated isoform has a premature stop codon.

Early studies on the characterisation of the murine GMRα failed to detect alternatively spliced isoforms [15] but a soluble receptor able to block GM-CSF function has been identified for the human counterpart [17]. Using cDNA from bone marrow cells, we identified full-length transcripts with open reading frame of 1167 bp (388 amino acids) for Balb/c and C57BL/6 mice, which are polymorphic at amino acid residue 227 (Asp and Gly, respectively) (Fig. 1A, B). We also found alternative splice forms in C57BL/6: a deletion isoform (▵223–247), which lacks part of the FN-III domain, and a truncated isoform that would be predicted to be soluble due to the absence of a transmembrane domain (Fig. 1). These sequences have been submitted to GenBank (accession numbers: EF114317 and EF114318).

The genomic structure of the mouse GMRα gene, *csf2ra*, has only recently been completed (mouse genome sequence Ensembl release 40). The transcripts we identified are shown schematically alongside this genomic sequence in Fig. 1A. The deletion isoform appears to be created by skipping of the splice acceptor of exon 6 and aberrant splicing into the coding sequence of exon 6. Similarly the truncation isoform is the result of inclusion of the fifth intron into the final transcript resulting in a premature stop codon (Fig. 1A).

### Functional analysis of polymorphic variants of the mouse GM-CSF receptor

We expressed full-length GMRα from both Balb/c and C57BL/6 mice in Ba/F3 cells, which constitutively express both AIC2A and AIC2B βc [14], permitting the generation of a functional αβ receptor, which should respond to GM-CSF. We cultured Ba/F3 cells (transduced with empty vector or GMRα-encoding vectors) in the presence of IL-3 or GM-CSF, and assessed cell proliferation daily by determining the percentage of reduction of Alamarblue, an oxidation-reduction indicator that changes color in response to metabolic activity [20].

**Figure 2:**
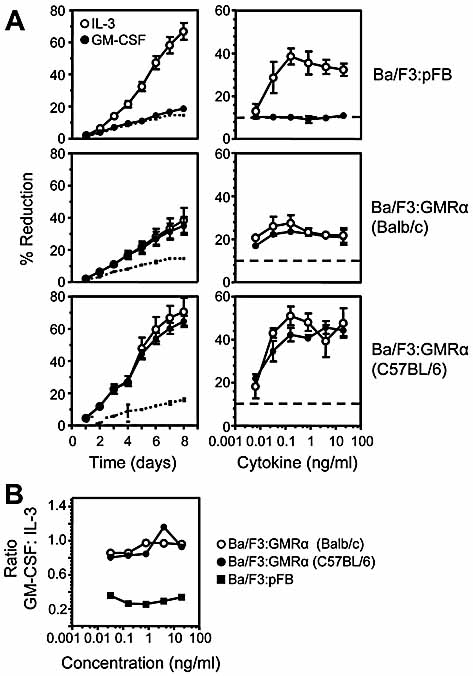
Functionality of polymorphic forms of GMRα. (A) Full-length GMRα from Balb/c and C57BL/6 mice were retrovirally transduced into Ba/F3 cells. Ba/F3 cells transduced with empty vector or GMRα-encoding vectors were cultured in the presence of 4 ng/mL of GM-CSF (filled circles) or IL-3 (open circles) for the indicated number of days (left panels) or in the indicated concentrations of cytokine for 8 days (right panels). Cell proliferation was measured by determining the percentage of reduction of Alamarblue. (B) The ratio of proliferation in response to GM-CSF and IL-3 was calculated to compare the polymorphic variants; filled squares: Ba/F3:pFB; open circles: Ba/F3:GMRα (Balb/c); filled circles: Ba/F3:GMRα (C57BL/6).

We found that empty vector-transduced control cells (Ba/F3:pFB), like untransduced Ba/F3 cells (data not shown), proliferated only in the presence of IL-3, but GMRα-transduced Ba/F3 cells (Ba/F3:GMRα) could proliferate in either IL-3 or GM-CSF (Fig. 2A). Using these conditions, we were able to detect significant proliferation of Ba/F3:GMRα after 5 days in culture with a concentration of GM-CSF as low as 0.032 ng/mL. Since we did not have a reagent to monitor expression of the polymorphic forms of GMRα, we calculated a ratio of proliferation to compare the functional activity of the polymorphic variants of GMRα in response to both GM-CSF and IL-3 (Fig. 2B). We found a similar proliferation ratio for both polymorphic forms of GMRα, indicating that the polymorphism does not affect responsiveness of the cell to GM-CSF.

### A novel GM-CSF chimeric protein and assessment of its functionality

**Figure 3:**
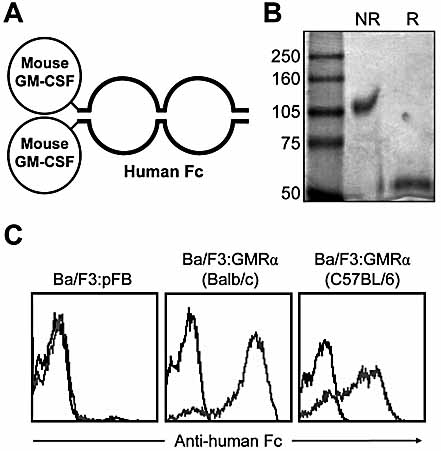
Generation and characterisation of a GM-CSF-Fc fusion protein. (A) Graphic representation of GM-Fc, a chimeric protein generated by fusing the mouse GM-CSF to a mutated Fc region of human IgG1. (B) GM-Fc purity was assessed by Coomassie blue staining of protein resolved under both reducing (R) and non-reducing (NR) conditions by 10% SDS-PAGE. (C) The binding capacity of GM-Fc (grey line) to Ba/F3 cells expressing GMRα from Balb/c or C57BL/6 mice was determined by FACS as indicated previously and compared to the control-Fc protein (black line).

The lack of antibodies against the extracellular domain of mouse GMRα has greatly restricted the study of this receptor. In order to circumvent this limitation, we designed a chimeric protein fusing the mouse GM-CSF to a mutated Fc region of human IgG1 (GM-Fc) (Fig. 3A, B), which contains point mutations to avoid complement activation and Fc receptor binding [21]. To evaluate the functionality of GM-Fc, we first determined its capacity to recognise Ba/F3 cells expressing GMRα (Fig. 3C). GM-Fc was able to bind the two polymorphic variants of GMRα (Balb/c and C57BL/6) while binding to cells transduced with empty vector was not detected.

**Figure 4:**
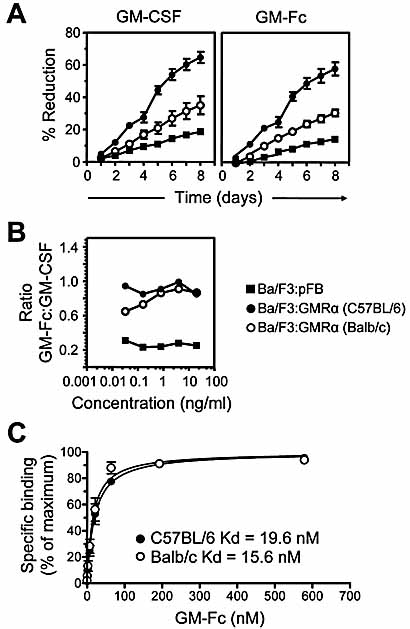
Effect of GM-Fc on Ba/F3 cells expressing full-length GMRα isoforms. (A) Ba/F3:pFB (filled squares), Ba/F3:GMRα (Balb/c; open circles), and Ba/F3:GMRα (C57BL/6; filled circles) were cultured in the presence of either GM-CSF or GM-Fc and cell proliferation was determined using Alamarblue as indicated previously. (B) Expressing percentage Alamarblue reduction obtained when GM-Fc or GM-CSF was used as a growth factor as a ratio shows that both agents have a similar capacity to support proliferation of Ba/F3:GMRα cells. (C) The ability of the C57BL/6 and Balb/c isoforms of GMRα to bind to the cytokine was assessed using GM-Fc and analysed by non-linear regression. Data shown represent pooled data from two independent experiments, and error bars shown the SEM.

We next cultured Ba/F3 cells (Ba/F3:pFB and Ba/F3:GMRα) in presence of either GM-CSF or GM-Fc and assessed cell proliferation as indicated above (Fig. 4A). Similarly to GM-CSF, GM-Fc was effective at inducing proliferation of Ba/F3:GMRα (Fig. 4B). To determine whether the polymorphism between C57BL/6 and Balb/c mice affected binding of the cytokine, we measured the binding of the recombinant protein to both transduced cell lines. There was no marked difference in the binding between the Balb/c (KD = 15.6 nM, 95%-confidence interval: 10.9–20.3) and C57BL/6 (KD = 19.6 nM, 95%-confidence interval: 15.6–23.6) forms of the receptor.

### Functional activity of the alternative splice forms of GMRα

**Figure 5:**
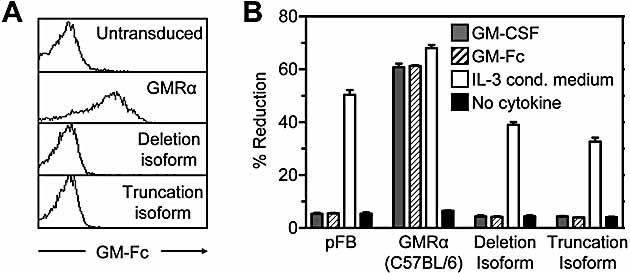
Effect of GM-Fc on Ba/F3 cells expressing the deletion and truncation GMRα isoforms. (A) The binding capacity of GM-Fc to Ba/F3 cells expressing the deletion and truncation GMRα from C57BL/6 mice was determined by FACS as indicated previously. (B) Ba/F3:pFB, Ba/F3:GMRα (C57BL/6), the deletion and truncation isoforms (C57BL/6) were cultured in the presence of either GM-CSF, GM-Fc, or IL-3-conditioned medium or in the absence of cytokine. Cell proliferation was determined using Alamarblue as indicated previously.

We next assessed the functional activity of the alternative splice forms of GMRα. Ba/F3 cells, stably transduced with the deletion and truncation isoforms of GMRα, were unable to bind GM-Fc (Fig. 5A). We verified this by culturing Ba/F3 cells expressing either the deletion or the truncation GMRα isoform in the presence of IL-3, GM-CSF or GM-Fc. Only those cells expressing the full-length GMRα proliferated in the presence of GM-CSF or GM-Fc. The cells expressing the deletion or truncation isoforms were only able to proliferate in the presence of IL-3 (Fig. 5B).

### Detection of GMRα on the surface of primary leukocytes

**Figure 6:**
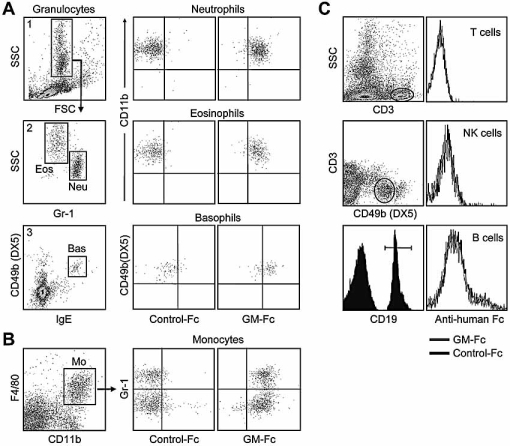
Detection of GMRα on the surface of blood leukocytes. Cell surface expression of mouse GMRα in different blood cell populations was determined by FACS using GM-Fc or control-Fc. (A) Granulocytes were gated by their SSC^high^FSC^int–high^ phenotype (left panel 1), and subdivided into neutrophils (Neu, Gr-1^high^SSC^high^; left panel 2), eosinophils (Eos, Gr-1^low^SSC^v.high^; left panel 2) and basophils (Bas, CD49b^+^IgE^+^; left panel 3). Binding of control-Fc (middle panels) and GM-Fc (right panels) was then assessed on the three populations as indicated. GM-Fc bound to all three granulocyte populations (B) Monocytes (Mo) were identified by their F4/80^+^CD11b^+^ phenotype (left panel) after gating on SSC^low^ cells (not shown). The monocytes were further divided by their expression of Gr-1 and assessed for binding of control-Fc (middle panel) or GM-Fc (right panel). GM-Fc bound well to both major monocyte subsets. (C) GMRα expression was also determined on lymphoid cells. T cells (top panels, gated on CD3^+^), NK cells (middle panels, gated on CD3^–^CD49b^+^) and B cells (bottom panels, gated on CD19^+^) were assessed for binding of GM-Fc (grey lines) and control-Fc (black line). No specific binding of GM-Fc was detected on the lymphocyte populations.

Cell surface expression of GMRα has been broadly studied in human cells [22], but little is known about its expression in the mouse model. We used GM-Fc to detect GMRα in different cell populations in blood, bone marrow and spleen. A detailed analysis of GMRα expression by the cell populations in peripheral blood is presented in Fig. 6. All populations of granulocytes (Gr-1^high^SSC^high^F4/80^–^ neutrophils, Gr-1^low^SSC^v.high^F4/80^+^ eosinophils and CD49b^+^IgE^+^SSC^high^ basophils) as well as Gr-1^+^ and Gr-1^–^ monocytes (SSC^low^F4/80^+^CD11b^+^) expressed similar levels of GMRα, with monocytes expressing the highest levels (Fig. 6A, B), while T cells (CD3^+^), NK cells (CD3^–^CD49b^+^) and B cells (CD19^+^) lacked GMRα expression (Fig. 6C). The control-Fc chimeric protein (CR^W117A^-Fc) was unable to bind either myeloid or lymphoid cells confirming the specificity of GM-Fc binding. The expression data from spleen and bone marrow are compared to those of blood and summarised in Table 1. Consistent with the expression pattern reported for the human homologue, we found that myeloid cells but not lymphoid cells expressed GMRα (Fig. 6 and Table 1).

**Table 1:** Summary of the expression of GMRα by mouse leukocytes

Tissue^a^)					
Cell type	Phenotype	Blood	Spleen	Bone marrow	Peritoneal cavity
**Monocytes**^b^)	SSC^low^CD11b^+^F4/80^+^	+	+	+	ND
**Macrophages**^c^)	F4/80^high^	ND	+	ND	+
Neutrophils	SSC^high^CD11b^+^Gr-1^high^	+	+	+	+
Eosinophils	SSC^v.high^CD11b^+^Gr-1^low^	+	+	+	+
Basophils	SSC^med^CD49b^+^IgE^+^	+	ND	ND	ND
**Dendritic cells**^d^)	CD11c^high^	ND	+	ND	+
T cells	CD3^+^	–	–	–	ND
B cells	CD19^+^	–	–	–	–
NK cells	CD3^–^CD49b^+^	–	–	–	ND

a + denotes GMRα expression; – denotes absence of detectable GMRα expression; ND denotes not detectable or not determined.

b Refers collectively to both Gr-1^+^ and Gr-1^–^ monocytes.

c Splenic macrophages are identified by autofluorescence and F4/80 expression, whereas peritoneal macrophages also express high levels of CD11b.

d Dendritic cell-like cells in the peritoneal cavity express relatively low levels of CD11c.

### Expression of GMRα on macrophages

**Figure 7:**
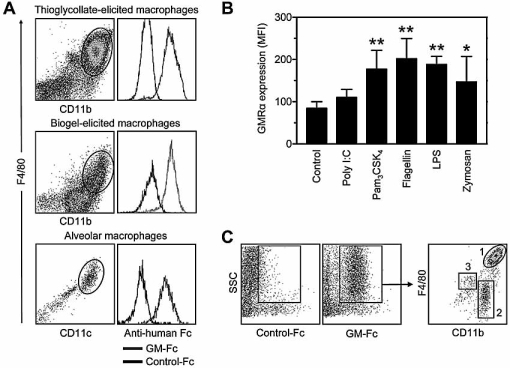
Expression of GMRα by macrophages and characterisation of myeloid cells. (A) Inflammatory peritoneal macrophages were elicited by intraperitoneal administration of thioglycollate broth (top panels) or biogel (middle panels) for 4 days. GMRα expression was analysed by FACS along with the macrophages identified by F4/80 and CD11b profiles (gated regions). Alveolar macrophages were isolated from lung lavage and gated in accordance with the expression of F4/80 and CD11c (bottom panels). GM-Fc (right panels, grey lines), when compared to control-Fc (right panels, black lines), bound specifically and at high levels to the macrophage populations. (B) RAW264.7 cells were stimulated with 100 ng/mL poly(I:C), Pam_3_CSK_4_, flagellin or LPS or 5×10^6^ particles/well of zymosan. After 24 h, binding of GM-Fc was assessed by FACS. Data represent the results of four independent experiments and error bars are the SD. Data were analysed by repeated measures one-way ANOVA with Dunnet's post test; *p<0.05, **p<0.01. (C) We used GM-Fc to identify GMRα-expressing cells in the resting peritoneal cavity (upper panels). Gating on GMRα^+^ cells identified three populations of myeloid cells, which expressed distinct levels of the myeloid markers CD11b and F4/80 (populations 1–3, upper right panel). The physical FSC/SCC profiles of three populations are shown in the lower panels. Population 1 exhibits the classic F4/80^high^CD11b^high^ phenotype of resident peritoneal macrophages. The second F4/80^low^CD11b^high^ population also expressed MHC class II and exhibited dendritic cell-like characteristics (Dioszeghy *et al*., manuscript in preparation) and the third rarer population shared the phenotype of eosinophils.

Since GM-CSF plays an important role in the production and function of macrophages, we analysed the expression of GMRα on inflammatory-elicited peritoneal macrophages and on alveolar macrophages. Macrophages elicited by intraperitoneal administration of thioglycollate broth or biogel (Fig. 7A) exhibited high levels of GMRα, as did alveolar macrophages (Fig. 7A). Injection of thioglycollate broth or biogel recruited neutrophils and eosinophils. These cells also expressed GMRα but the levels of receptor were lower than in the inflammatory macrophages (data not shown).

To determine changes in the expression of GMRα following stimulation, we treated RAW264.7 cells (a mouse macrophage-like cell line expressing high levels of GMRα on their cell surface) with a range of inflammatory insults and assessed binding of GM-Fc. We found that RAW264.7 cells selectively up-regulated GMRα surface expression in response to a variety of Toll-like receptor agonists, including Pam_3_CSK_4_, flagellin, lipopolysaccharide (LPS) and zymosan, but not to poly(I:C) (Fig. 7B).

### Expression of GMRα as a marker of myeloid cells

The restriction of GMRα to cells of the myeloid, but not lymphoid, lineage indicated that GMRα may be a useful marker of myeloid cells in the mouse. The peritoneal cavity contains a variety of immune cells, which have traditionally been considered as the first line of defence against invasion of micro-organisms in the peritoneum [23]. We used GM-Fc to identify GMRα-expressing cells in the resting peritoneal cavity, and the positive population was further analysed by expression of the additional myeloid markers F4/80 and CD11b. The peritoneal GMRα^–^ cells corresponded predominantly to lymphocytes, mainly B cells (data not shown).

We were able to clearly differentiate three distinct populations (Fig. 7C). The most common population representing about 30–40% of peritoneal cells had the classic phenotype of resident peritoneal macrophages (CD11b^high^F4/80^high^, population 1 in Fig. 7C). A sparse population, which expressed moderate levels of both CD11b and F4/80, had high SSC and shared additional markers with eosinophils (Fig. 7C and data not shown). Most interesting was the identification of an apparently novel cell type, which represented between 1 and 5% of the peritoneal cells depending on mouse strain and had a CD11b^high^, but F4/80^low^ phenotype. We subsequently purified and began the functional characterisation of these cells. They express high levels of MHC class II and can present antigen to ovalbumin-specific CD4^+^ T cells from naive DO11.10 mice indicative of a dendritic cell-like phenotype (Dioszeghy *et al*., manuscript in preparation).

## Discussion

The structure and function of the human GMRα has been broadly investigated, and several studies using mice lacking either GM-CSF [24], [25] or the βc of its receptor [26], [27], as well as administration of GM-CSF [28–30] or neutralising antibodies against the cytokine [31], [32] have been used in models of disease. However, fundamental knowledge about the nature of expression and function of the mouse GMRα is lacking.

In this study, we cloned GMRα-coding sequences from Balb/c and C57BL/6 mice. We found that mouse GMRα is polymorphic at amino acid residue 227 (Asp and Gly, respectively) and alternatively spliced. As well as amplifying full-length clones, we identified two alternatively spliced isoforms that have not been previously described. These alternatively spliced isoforms were expressed in bone marrow cells, where the predominant expression of GMRα is by neutrophils and monocytes (Table 1). We retrovirally transduced the IL-3-dependent AIC2A/AIC2B-expressing Ba/F3 cell line with the full-length GMRα clones from Balb/c or C57BL/6 mice. The Ba/F3:GMRα cells were able to proliferate in response to GM-CSF, unlike the control vector-transduced cells, and comparative analysis of the responses to cell lines to IL-3 and GM-CSF indicated that the polymorphism did not significantly affect receptor function. Apart from the polymorphic residue mentioned above, our sequences were identical to the published cDNA [15].

Since there are no commercial reagents for the detection of mouse GMRα at the cell surface, we designed a chimeric protein (GM-Fc) which had mouse GM-CSF fused to the Fc domain of human IgG1 (Fig. 3). GM-Fc bound specifically to GMRα-transduced Ba/F3, but not control transduced cells, confirming its specificity as a receptor-detecting reagent. However, GM-Fc was not suitable for immunohistochemical analysis of the expression of GMRα in fixed tissue sections, most likely because of the need for heterodimeric receptor for high-affinity binding. GM-Fc was also able to support the proliferation of the GMRα-transduced Ba/F3 cells, indicating that the reagent functioned in a comparable way to the natural cytokine making possible its implementation in functional studies *in vitro* and *in vivo*. GM-Fc was thus a specific and sensitive immunological tool to study the expression of the murine GMRα on a variety of cell types.

One of the mouse GMRα isoforms lacked the transmembrane region (truncated isoform, Fig. 1), which by analogy to the human receptor isoforms could be secreted into the extracellular environment as a soluble receptor capable of antagonizing receptor functions [16], [17]. Consistent with this we were unable to detect this isoform on the surface of transduced cells with GM-Fc (Fig. 5A). The second isoform contained a deletion (▵223–247) within the FN-III domain, but had an intact transmembrane domain and would be predicted to be expressed at the cell surface (Fig. 1). We were unable to detect GM-Fc binding on cells transduced to express this isoform, suggesting that it was incapable of cytokine binding (Fig. 5A). The biological relevance of these isoforms remains to be established but they could be significant in the regulation of GMRα expression and in the study of diseases associated with deficiency of GM-CSF:GM-CSF receptor, such as pulmonary alveolar proteinosis [33].

We have used GM-Fc for the first sensitive assessment of the surface expression of GMRα on primary leucocytes derived from different mouse tissues. Similarly to human cells [34], macrophages [alveolar and peritoneal, both resident and inflammatory (thioglycollate- or biogel-elicited)], were the highest GMRα- expressing cells followed by both Gr-1^+^ and Gr-1^–^ monocytes (from blood, spleen and bone marrow). Neutrophils and eosinophils (from blood, spleen, bone marrow and peritoneal lavage) as well as circulating basophils expressed reasonable levels of GMRα but less than those expressed by macrophages or dendritic cells. Lymphoid cells (B cells, T cells and NK cells) derived from blood, spleen and bone marrow did not express GMRα. In the same way, human lymphocytes (B cells, T cells and NK cells) do not express GMRα but its presence has been demonstrated in some malignancies like the hairy-cell leukaemia, breast and lung carcinomas as well as on several hemopoietic cell lines [35–37]. These data are also consistent with studies on hematopoietic commitment and differentiation, which have revealed that GMRα expression contributes to the development of myeloid cells while its expression impacts negatively on the lymphoid lineage [38], [39].

We were unable to detect expression on non-hemopoietic cell lines such as b.END-3, NIH3T3 and, perhaps more interestingly given the role of GM-CSF in the lung homeostasis, LA-4 respiratory epithelial cells (data not shown). We extended these studies by applying GM-Fc to the study of the macrophage inflammatory response by determining changes in the surface expression of GMRα in response to microbial stimuli. The Toll-like receptor agonists Pam_3_CSK_4_, flagellin, zymosan and LPS all induced an up-regulation of GMRα surface expression, validating the use of GM-Fc as a sensitive way to monitor the regulation of GMRα expression by immune cells.

We were interested in the resident cells dwelling in the peritoneal cavity, which are thought to be essential in the local defensive response against infection [23]. The peritoneal cavity has been extensively exploited to study inflammation but the resident myeloid cells have not been well characterised. We defined three populations of GMRα-expressing cells in the peritoneal cavity by their CD11b and F4/80 profiles (Fig. 7C). The major population, representing 30–40% of the normal peritoneal cells, had the classic phenotype of peritoneal macrophages [40]. We also identified a second population (CD11b^high^F4/80^low^), not previously described, which after further functional analysis appears to comprise dendritic-like cells, as determined by the expression of MHC class II and ability to present antigen to naive T cells (Dioszeghy *et al*., manuscript in preparation). Several studies have suggested the presence of dendritic cells in the peritoneal cavity but they have demonstrated a capacity to demonstrate antigen only after *in vitro* differentiation [30], [41], [42]. The third and rarest population shared an antigen phenotype with eosinophils (data not shown) [43].

Taken together, this characterisation of the mouse GMRα as well as its expression on leukocytes from different tissues provides fundamental information that can be applied to many immunological studies. The receptor is polymorphic and importantly alternatively spliced, indicating that the regulation of its function in mouse models of disease may be as complex as its human counterpart. In the absence of antibodies, GM-Fc provides a sensitive and specific tool for the study of murine GMRα and its regulation during immunological challenge.

## Materials and methods

### Mice and reagents

Balb/c and C57BL/6 mice were obtained from our own breeding colonies, kept and handled in accordance with institutional and UK Home Office guidelines. Mouse recombinant GM-CSF and IL-3 were obtained from R&D Systems, Inc. (Minneapolis, MN). IL-3-conditioned medium was derived by culture of IL-3-producing WEHI-3 cells in RPMI 1640 (Gibco, UK) supplemented with 10% heat-inactivated fetal calf serum (FCS). RAW264.7, b.END-3, NIH3T3 and LA-4 cells (a gift from Dr. T. Tuthill [44]), were used as models of macrophages, endothelial cells, fibroblasts and respiratory epithelial cells, respectively. They were cultured in DMEM supplemented with 10% FCS, 2 mM l-glutamine, penicillin and streptomycin (b.END-3 cells were also supplemented with 1% w/v non-essential amino acids, 5 µM 2-mercaptoethanol and 1 mM sodium pyruvate). Anti-IgE labelled with fluorescein isothiocyanate (FITC), anti-CD19 labelled with R-phycoerythrin (PE), and anti-F4/80-PE antibodies were purchased from Serotec (Oxford, UK). Anti-CD49b (DX5)-PE and anti-Gr-1-PE were obtained from BD Pharmingen (Franklin Lakes, NJ). Anti-CD3-FITC was from Caltag Medsystems (Buckingham, UK). Anti-CD11b-FITC (clone 5C6) [45] and anti-CD11c (clone N418) [46] were produced in our laboratory. Anti-human-Fc labelled with allophycocyanin or PE were purchased from Jackson Immunoresearch Laboratories, Inc.

### Generation of GMRα-stable cell lines

RNA was isolated from bone marrow cells of both Balb/c and C57BL/6 mice according to standard conditions of the RNAeasy spin protocol (Qiagen). cDNA synthesis was performed using the Advantage RT-PCR kit (BD Biosciences) and GMRα was amplified by PCR with the following primers (Sigma): AGAGGAATTCCACCATGACGTCATCACATGCCATG-5′ and AGAGGAATTCTAGGGCTGCAGGAGGTCCTT-3′. PCR products were cloned into pCR3.1 vector (Invitrogen Life Technologies) and sequenced (Sir William Dunn School of Pathology sequencing service). GMRα isoforms were further subcloned into the retroviral vector pFB(neo) (Stratagene) and transfected into the HEK293T-based Phoenix ecotropic packaging cells using FuGene 6 (Roche). Retroviral supernatants were collected after 48 h and used to transduce Ba/F3 cells, a murine IL-3-dependent pro-B cell line. Stable cell lines were selected and maintained in 0.6 mg/mL geneticin (Sigma-Aldrich).

### Proliferation assay

Ba/F3 cells transduced with either empty vector, the full-length isoforms (Balb/c or C57BL/6), or the deletion or truncation isoforms (C57BL/6) of the GMRα were cultured in RPMI 1640 (Gibco) supplemented with 10% heat-inactivated FCS and the indicated concentration of cytokines (GM-CSF or IL-3). Experiments were performed in 96-well plates using an initial cell density of 1000 cells per well in a final volume of 200 μL with 10% v/v Alamarblue (Serotec). Absorbance was measured at a wavelength of 570 nm and 600 nm daily during 8 days. Percentage reduction of Alamarblue as a measure of proliferation was determined according to the manufacturer's instructions.

### Generation of GM-CSF-Fc

GM-CSF was amplified by PCR using the following primers (Sigma): GGGGAATTCATGTGGCTGCAGAATTTACTTTTCCT-5′ and GGGGAATTCTTTTTGGCCTGGTTTTTTGCATTCAAA-3′. The PCR product was digested with *Eco*RI and cloned in the correct reading frame of a pSecTag2(C) vector (Invitrogen, UK), which contains the Igκ signal peptide and was previously modified by the insertion of a human IgG1 Fc domain with its own stop codon [21]. This Fc domain contains four mutations that remove Fc receptor binding and complement activation [21]. The resultant vector encoded a GM-CSF-Fc chimeric protein (referred to as GM-Fc) under the control of the CMV promoter. GM-Fc was transfected into HEK293T cells using GeneJuice (Novagen, UK), and stable clones producing GM-Fc were selected in DMEM (Gibco) containing 4% IgG-depleted bovine serum and 0.2 mg/mL zeocin (Invitrogen Life Technologies).

GM-Fc was also subcloned into the *BamHI* restriction site of pFB(neo) (Stratagene) subsequent to PCR amplification of GM-Fc using the following primers: GGAGGATCCACCATGTGGCTGCAGAATTTACTT-5′ and GGAGGATCCTCATTTACCCGGAGACAGGG-3′. This construct was transfected into HEK293T-based Phoenix ecotropic packaging cells and the retrovirus-containing medium was used to transduce NIH3T3 cells as previously described. Stable cell lines were selected and maintained in DMEM (Gibco) containing 4% IgG-depleted bovine serum and 0.6 mg/mL geneticin (Sigma-Aldrich).

HEK293T or NIH3T3 cells expressing GM-Fc were left to condition media (above) for at least 7 days. GM-Fc was isolated by affinity purification on Protein A-sepharose (GE Healthcare) and eluted with 0.1 M glycine, pH 2.9. After neutralisation with 0.1 volume of 1 M Tris, pH 9.5, GM-Fc was dialysed against PBS and protein concentration determined by the bicinchoninic acid method (Pierce). The purity of individual batches of GM-Fc was assessed by Coomassie blue staining of protein resolved under both reducing and non-reducing conditions by 10% SDS-PAGE. To establish that binding of GM-Fc was specific, CR^W117A^-Fc was utilized as control-Fc protein since it contains a mutated version of the cysteine-rich domain (CR) of the mannose receptor that abrogates lectin activity and lacks binding capacity [21].

### FACS experiments and analysis

The binding of GM-Fc to GMRα-expressing cells (GMRα- transduced Ba/F3 cells or cells derived from blood, spleen, bone marrow as well as alveolar and peritoneal lavage) was determined by FACS. Briefly, cells were incubated in blocking buffer 1 (PBS containing 5% heat-inactivated rabbit serum, 0.5% BSA, 5 mM EDTA and 2 mM NaN_3_) for 1 h at 4°C. GM-Fc or control-Fc were added at 10 μg/mL in a final volume of 100 μL of washing buffer (PBS containing 0.5% BSA, 5 mM EDTA and 2 mM NaN_3_), and incubated for 1 h at 4°C. After two washes, cells were incubated with anti-human-allophycocyanin and washed again 1 h later. Cells were incubated in blocking buffer 2 (PBS containing 5% heat-inactivated rat serum, 0.5% BSA, 5 mM EDTA and 2 mM NaN_3_) for 1 h at 4°C. This step was necessary to block residual anti-rat activity of the anti-human reagent before adding labelled rat anti-mouse antibodies for the specific markers [IgE, CD19, F4/80, CD49b (DX5), Gr-1, CD3 and CD11b].

After three washes, cells were resuspended in 1% formaldehyde (in PBS) and acquired on a FACScalibur (Becton Dickinson). FACS analysis was performed using FlowJo (Tree Star, Inc.). Granulocytes were gated on a FSC/SSC plot and sorted using specific markers: neutrophils (SSC^med^CD11b^+^ Gr-1^high^), eosinophils (SSC^high^CD11b^+^Gr-1^low^) and basophils (SSC^med^CD49b^+^IgE^+^). Monocytes (SSC^med^CD49b^+^IgE^+^) were subdivided into GR-1^+^ and Gr-1^–^ subsets, and lymphocytes were classified as T cells (CD3^+^), B cells (CD19^+^) and NK cells (CD3^–^CD49b^+^).

### Stimulation of RAW264.7 cells

RAW264.7 cells were seeded on 24-well plates at a density of 50 000 cells per well in DMEM supplemented with 10% FCS, l-glutamine, penicillin and streptomycin. Next day, the medium was removed and cells were cultured in presence of 100 ng/mL of poly(I:C), Pam_3_CSK_4_, flagellin, LPS or zymosan at 5×10^6^ particles per well. Cells were incubated at 37°C for 24 h in presence of the stimuli. After this time, cells were washed with PBS and incubated with blocking buffer (PBS containing 5% heat-inactivated rabbit serum, 0.5% BSA and 2 mM NaN_3_) for 1 h at 4°C. Cells were stained *in situ* using GM-Fc or control-Fc (as described above). After staining, the cells were lifted with a scraper and transferred to a FACS tube in 1% formaldehyde. FACS acquisition and analysis were performed as indicated above. The data from four independent experiments were analysed for statistically significant differences by one-way repeated measures ANOVA test with Dunnett's multiple comparison test using GraphPad Prism.

### Analysis of the binding of GM-Fc to GMRα isoforms

For analysis of the binding efficiency of GM-Fc to the polymorphic variants of GMRα, a range of concentrations of GM-Fc were incubated with GMRα-transduced Ba/F3 cells for 2 h at 4°C. The cells were washed and the bound protein was detected as described above using anti-human-allophycocyanin and flow cytometry. Specific binding was determined by subtraction of the non-specific binding of GM-Fc to control pFBneo-transduced Ba/F3 cells. Binding data were analysed by non-linear regression using GraphPad Prism.

## Abbreviations:

βc: β-chain of the GM-CSF receptor
FN-III: fibronectin type III
GM-Fc: mouse GM-CSF-human IgG1 Fc fusion protein
GMRα: GM-CSF receptor α-chain

## References

[1] Hamilton JA, Anderson GP (2004). GM-CSF biology. Growth Factors.

[2] Armitage JO (1998). Emerging applications of recombinant human granulocyte-macrophage colony-stimulating factor. Blood.

[3] Trapnell BC, Whitsett JA (2002). GM-CSF regulates pulmonary surfactant homeostasis and alveolar macrophage-mediated innate host defense. Annu. Rev. Physiol..

[4] Reed JAH, Rice WR, Zsengeller ZK, Wert SE, Dranoff G, Whitsett JA (1997). GM-CSF enhances lung growth and causes alveolar type II epithelial cell hyperplasia in transgenic mice. Am. J. Physiol. Lung Cell. Mol. Physiol..

[5] Wognum AW, Westerman Y, Visser TP, Wagemaker G (1994). Distribution of receptors for granulocyte-macrophage colony-stimulating factor on immature CD34^+^ bone marrow cells, differentiating monomyeloid progenitors, and mature blood cell subsets. Blood.

[6] Chegini N, Tang X.-M, Dou Q (1999). The expression, activity and regulation of granulocyte macrophage-colony stimulating factor in human endometrial epithelial and stromal cells. Mol. Hum. Reprod..

[7] Soldi R, Primo L, Brizzi MF, Sanavio F, Aglietta M, Polentarutti N, Pegoraro L (1997). Activation of JAK2 in human vascular endothelial cells by granulocyte-macrophage colony-stimulating factor. Blood.

[8] Dhar-Mascareno M, Pedraza A, Golde DW (2005). PI3-kinase activation by GM-CSF in endothelium is upstream of JAK/STAT pathway: Role of αGMR. Biochem. Biophys. Res. Commun..

[9] Postiglione L, Montagnani S, Riccio A, Ladogana P, Salzano S, Vallefuoco L, Rossi G (1998). Expression of GM-CSF receptor and *in vitro* effects of GM-CSF on human fibroblasts. Life Sci..

[10] Barreda DR, Hanington PC, Belosevic M (2004). Regulation of myeloid development and function by colony stimulating factors. Dev. Comp. Immunol..

[11] Guthridge MA, Stomski FC, Thomas D, Woodcock JM, Bagley CJ, Berndt MC, Lopez AF (1998). Mechanism of activation of the GM-CSF, IL-3, and IL-5 family of receptors. Stem Cells.

[12] de Groot RP, Coffer PJ, Koenderman L (1998). Regulation of proliferation, differentiation and survival by the IL-3/IL-5/GM-CSF receptor family. Cell Signal..

[13] Itoh N, Yonehara S, Schreurs J, Gorman DM, Maruyama K, Ishii A, Yahara I (1990). Cloning of an interleukin-3 receptor gene: A member of a distinct receptor gene family. Science.

[14] Kitamura T, Hayashida K, Sakamaki K, Yokota T, Arai K, Miyajima A (1991). Reconstitution of functional receptors for human granulocyte/macrophage colony-stimulating factor (GM-CSF): Evidence that the protein encoded by the AIC2B cDNA is a subunit of the murine GM-CSF receptor. Proc. Natl. Acad. Sci. USA.

[15] Park LS, Martin U, Sorensen R, Luhr S, Morrissey PJ, Cosman D, Larsen A (1992). Cloning of the low-affinity murine granulocyte-macrophage colony-stimulating factor receptor and reconstitution of a high-affinity receptor complex. Proc. Natl. Acad. Sci. USA.

[16] Raines MA, Liu L, Quan SG, Joe V, DiPersio JF, Golde DW (1991). Identification and molecular cloning of a soluble human granulocyte-macrophage colony-stimulating factor receptor. Proc. Natl. Acad. Sci. USA.

[17] Williams WV, VonFeldt JM, Rosenbaum H, Ugen KE, Weiner DB (1994). Molecular cloning of a soluble form of the granulocyte-macrophage colony-stimulating factor receptor alpha chain from a myelomonocytic cell line. Expression, biologic activity, and preliminary analysis of transcript distribution. Arthritis Rheum..

[18] Brown CB, Beaudry P, Laing TD, Shoemaker S, Kaushansky K (1995). *In vitro* characterization of the human recombinant soluble granulocyte-macrophage colony-stimulating factor receptor. Blood.

[19] Prevost JM, Pelley JL, Zhu W, D'Egidio GE, Beaudry PP, Pihl C, Neely GG (2002). Granulocyte-macrophage colony-stimulating factor (GM-CSF) and inflammatory stimuli up-regulate secretion of the soluble GM-CSF receptor in human monocytes: Evidence for ectodomain shedding of the cell surface GM-CSF receptor alpha subunit. J. Immunol..

[20] Ahmed SA, Gogal RM, Walsh JE (1994). A new rapid and simple non-radioactive assay to monitor and determine the proliferation of lymphocytes: An alternative to [^3^H]thymidine incorporation assay. J. Immunol. Methods.

[21] Taylor PR, Zamze S, Stillion RJ, Wong SY, Gordon S, Martinez-Pomares L (2004). Development of a specific system for targeting protein to metallophilic macrophages. Proc. Natl. Acad. Sci. USA.

[22] Gasson JC (1991). Molecular physiology of granulocyte-macrophage colony-stimulating factor. Blood.

[23] Broche F, Tellado JM (2001). Defense mechanisms of the peritoneal cavity. Curr. Opin. Crit. Care.

[24] Stanley E, Lieschke GJ, Grail D, Metcalf D, Hodgson G, Gall JAM, Maher DW (1994). Granulocyte/macrophage colony-stimulation factor-deficient mice show no major perturbation of hematopoiesis but develop a characteristic pulmonary pathology. Proc. Natl. Acad. Sci. USA.

[25] Wada H, Noguchi Y, Marino MW, Dunn AR, Old LJ (1997). T cell functions in granulocyte/macrophage colony-stimulating factor deficient mice. Proc. Natl. Acad. Sci. USA.

[26] Nishinakamura R, Miyajima A, Mee PJ, Tybulewicz VL, Murray R (1996). Hematopoiesis in mice lacking the entire granulocyte-macrophage colony-stimulating factor/interleukin-3/interleukin-5 functions. Blood.

[27] Reed JA, Ikegami M, Robb L, Begley CG, Ross G, Whitsett JA (2000). Distinct changes in pulmonary surfactant homeostasis in common beta-chain- and GM-CSF-deficient mice. Am. J. Physiol. Lung Cell. Mol. Physiol..

[28] Metcalf D, Begley CG, Williamson DJ, Nice EC, De Lamarter J, Mermod JJ, Thatcher D, Schmidt A (1987). Hemopoietic responses in mice injected with purified recombinant murine GM-CSF. Exp. Hematol.

[29] Pojda ZMG, Dexter TM (1989). Effects of long-term *in vivo* treatment of mice with purified murine recombinant GM-CSF. Exp. Hematol..

[30] Rezzani R, Rodella L, Zauli G, Caimi L, Vitale M (1999). Mouse peritoneal cells as a reservoir of late dendritic cell progenitors. Br. J. Haematol..

[31] Yamashita N, Tashimo H, Ishida H, Kaneko F, Nakano J, Kato H, Hirai K (2002). Attenuation of airway hyperresponsiveness in a murine asthma model by neutralization of granulocyte-macrophage colony-stimulating factor (GM-CSF). Cell. Immunol..

[32] Bozinovski S, Jones J, Beavitt S.-J, Cook AD, Hamilton JA, Anderson GP (2004). Innate immune responses to LPS in mouse lung are suppressed and reversed by neutralization of GM-CSF *via* repression of TLR-4. Am. J. Physiol. Lung Cell. Mol. Physiol..

[33] Seymour JF, Presneill JJ (2002). Pulmonary alveolar proteinosis: Progress in the first 44 years. Am. J. Respir. Crit. Care Med..

[34] Jubinsky PT, Laurie AS, Nathan DG, Yetz-Aldepe J, Sieff CA (1994). Expression and function of the human granulocyte-macrophage colony-stimulating factor receptor alpha subunit. Blood.

[35] Till KJ, Burthem J, Lopez A, Cawley JC (1996). Granulocyte-macrophage colony-stimulating factor receptor: Stage- specific expression and function on late B cells. Blood.

[36] Lee KY, Suh BG, Kim JW, Lee W, Kim SY, Kim YY, Lee J (2000). Varying expression levels of colony stimulating factor receptors in disease states and different leukocytes. Exp. Mol. Med..

[37] Dabusti M, Castagnari B, Moretti S, Ferrari L, Tieghi A, Lanza F (2001). CD116 (granulocyte-macrophage colony stimulating factor receptor). J. Biol. Regul. Homeost. Agents.

[38] Kondo M, Scherer DC, Miyamoto T, King AG, Akashi K, Sugamura K, Weissman IL (2000). Cell-fate conversion of lymphoid-committed progenitors by instructive actions of cytokines. Nature.

[39] Iwasaki-Arai J, Iwasaki H, Miyamoto T, Watanabe S, Akashi K (2003). Enforced granulocyte/macrophage colony-stimulating factor signals do not support lymphopoiesis, but instruct lymphoid to myelomonocytic lineage conversion. J. Exp. Med..

[40] Taylor PR, Brown GD, Geldhof AB, Martinez-Pomares L, Gordon S (2003). Pattern recognition receptors and differentiation antigens define murine myeloid cell heterogeneity *ex vivo*. Eur. J. Immunol..

[41] van Vugt E, Arkema JM, Verdaasdonk MA, Beelen RH, Kamperdijk EW (1991). Morphological and functional characteristics of rat steady state peritoneal dendritic cells. Immunobiology.

[42] Makala LH, Nishikawa Y, Kamada T, Xuan X, Nagasawa H (2001). Antigen presentation by murine peritoneal cavity macrophage-derived dendritic cells. Pathobiology.

[43] Hamaguchi-Tsuru E, Nobumoto A, Hirose N, Kataoka S, Fujikawa-Adachi K, Furuya M, Tominaga A (2004). Development and functional analysis of eosinophils from murine embryonic stem cells. Br. J. Haematol..

[44] Tuthill TJ, Papadopoulos NG, Jourdan P, Challinor LJ, Sharp NA, Plumpton C, Shah K (2003). Mouse respiratory epithelial cells support efficient replication of human rhinovirus. J. Gen. Virol..

[45] Rosen H, Gordon S (1987). Monoclonal antibody to the murine type 3 complement receptor inhibits adhesion of myelomonocytic cells *in vitro* and inflammatory cell recruitment *in vivo*. J. Exp. Med..

[46] Metlay JP, Witmer-Pack MD, Agger R, Crowley MT, Lawless D, Steinman RM (1990). The distinct leukocyte integrins of mouse spleen dendritic cells as identified with new hamster monoclonal antibodies. J. Exp. Med..

